# Artificial intelligence guidance of advanced heart failure therapies: A systematic scoping review

**DOI:** 10.3389/fcvm.2023.1127716

**Published:** 2023-02-24

**Authors:** Mohammad A. Al-Ani, Chen Bai, Amal Hashky, Alex M. Parker, Juan R. Vilaro, Juan M. Aranda Jr., Benjamin Shickel, Parisa Rashidi, Azra Bihorac, Mustafa M. Ahmed, Mamoun T. Mardini

**Affiliations:** ^1^Division of Cardiovascular Medicine, University of Florida, Gainesville, FL, United States; ^2^Department of Health Outcomes and Biomedical Informatics, University of Florida, Gainesville, FL, United States; ^3^Department of Computer and Information Science and Engineering, University of Florida, Gainesville, FL, United States; ^4^Department of Medicine, University of Florida, Gainesville, FL, United States; ^5^Intelligent Critical Care Center (IC^3^), University of Florida, Gainesville, FL, United States; ^6^Department of Biomedical Engineering, University of Florida, Gainesville, FL, United States

**Keywords:** artificial intelligence, machine learning, deep learning, heart transplantation, mechanical circulatory support, LVAD

## Abstract

**Introduction:**

Artificial intelligence can recognize complex patterns in large datasets. It is a promising technology to advance heart failure practice, as many decisions rely on expert opinions in the absence of high-quality data-driven evidence.

**Methods:**

We searched Embase, Web of Science, and PubMed databases for articles containing “artificial intelligence,” “machine learning,” or “deep learning” and any of the phrases “heart transplantation,” “ventricular assist device,” or “cardiogenic shock” from inception until August 2022. We only included original research addressing post heart transplantation (HTx) or mechanical circulatory support (MCS) clinical care. Review and data extraction were performed in accordance with PRISMA-Scr guidelines.

**Results:**

Of 584 unique publications detected, 31 met the inclusion criteria. The majority focused on outcome prediction post HTx (*n* = 13) and post durable MCS (*n* = 7), as well as post HTx and MCS management (*n* = 7, *n* = 3, respectively). One study addressed temporary mechanical circulatory support. Most studies advocated for rapid integration of AI into clinical practice, acknowledging potential improvements in management guidance and reliability of outcomes prediction. There was a notable paucity of external data validation and integration of multiple data modalities.

**Conclusion:**

Our review showed mounting innovation in AI application in management of MCS and HTx, with the largest evidence showing improved mortality outcome prediction.

## Introduction

Advanced heart failure therapies are complex interventions, including mechanical circulatory support (MCS) and heart transplantation (HTx). These treatments can be highly rewarding, restoring quality of life and longevity, however, they are associated with relatively high adverse risk profile. Additionally, the target population is heterogeneous in hemodynamic requirements and risk profile for pre- and post-intervention complications. Ethically such patients are difficult to randomize to therapies when common practice suggests a standard of care. Also, the time lag between innovation, scholarly investigation, and clinical practice significantly limits evidence to guide patient management. Artificial intelligence (AI) has the power and resilience to integrate patient data from several domains and help clinicians navigate the care of the advanced heart failure therapy patient.

As the fields of AI and heart failure therapy both evolve exponentially and in parallel, it remains unclear how AI can integrate in clinical practice and whether these methods are mature enough for clinical application. This scoping review aims to systematically summarize and appraise the literature available in this arena, under the following research question: can AI guide clinicians in personalizing the practice of HTx and MCS to optimize longevity, quality of life, and resource utilization?

## Methods

The protocol was performed according to Preferred Reporting Items for Systematic Reviews and Meta-Analyzes extension for Scoping Reviews (PRISMA-ScR) guidelines (1). We searched Embase, Web of Science, and PubMed databases for published articles containing any of the phrases “artificial intelligence,” “machine learning,” or “deep learning” and any of the phrases “heart transplantation,” “ventricular assist device,” or “cardiogenic shock.” The latter term was included to target the group on temporary mechanical circulatory support. Search criteria included the above terms anywhere in the title, abstract, or keywords without any filters. We excluded review articles, meta-analyzes, conference abstracts, non-English language, animal and *ex-vivo* studies, non-AI methods, and those whose primary outcome is in the pre-HTx or MCS phase of care. Methodology was considered “AI” based if it fell under the main categories of supervised learning, unsupervised learning, or reinforcement learning (2). The search was not restricted by the year of publication. However, the number of publications related to AI in medicine has increased exponentially since 2008 (3).

Full text review and data extraction of each article were performed by at least one HF and one AI specialist. Conflicts were resolved by a HF specialist (MA). Search results were exported to EndNote (version 20.4.1), where duplicates were automatically identified and removed. The Covidence platform was used for title and abstract screening, full text screening, and data extraction. As this is a scoping review with most studies being first of kind or proof of concept, we have not excluded studies based on quality. Also, the group is heterogenous in methodologies, making objective head–head quality assessment unfeasible. The strength of recommending the AI algorithm for clinical use was categorized based on the message conveyed to the reviewer by the article discussion and conclusion sections.

## Results

**Figure 1:**
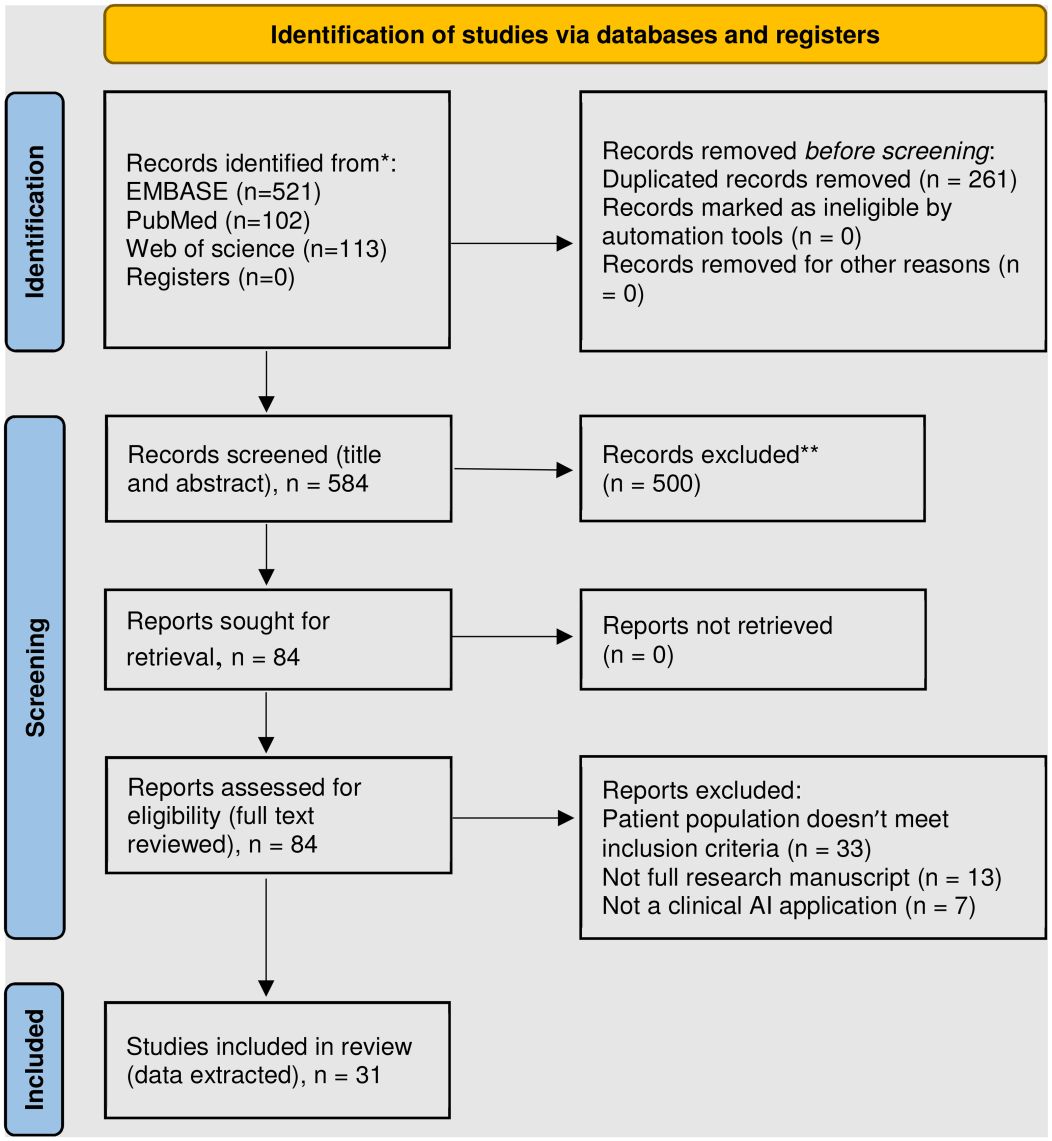
PRISMA 2020 flow diagram for new systematic reviews which included searches of databases and registers only (4). ^*^Consider, if feasible to do so, reporting the number of records identified from each database or register searched (rather than the total number across all databases). **Exclusion criteria included: review articles, meta-analyzes, conference abstracts, non-English language, animal and *ex-vivo* studies, non-AI methods, and those whose primary outcome is in the phase of care prior to transplantation or mechanical circulatory support.

Our search resulted in 584 publications, of which 17.5% were included in PubMed as many were published *via* biomedical informatics outlets that are not usually indexed in PubMed. Figure 1 summarizes study screening and exclusion reasons. A total of 31 manuscripts were included in our review, of which data were extracted and summarized from both clinical and informatics perspectives.

### Post heart transplant outcome prediction

We found 13 studies that used AI to predict post-HTx outcomes (Table 1). The most common data sources used for development, training, and validation of AI algorithms are the United for Organ Sharing (UNOS) and the International Society of Heart and Lung Transplantation (ISHLT) registry. Both data souces include massive numbers of HTx recipients and donors over four decades with a wide range of relevant donor and recipient variables of relatively high accuracy. While both data sources overcome limitations of generalizability of single center data, special challenges emerged when applying AI algorithms. The main challenge with UNOS data is the high number of missing values, requiring variable elimination and complex data imputation methods (5–7). The ISHLT registry, on the other hand, includes the UNOS database plus data from other centers worldwide – recently over 350 entities contributing (8). The ISHLT registry does not include wait list duration or mortality (9, 10). In addition, the data reporting varied between regions, centers, and eras. This could introduce systematic difference between training, testing, and validation datasets, thus confounding algorithm development (11). Algorithms applied to local datasets showed much higher performance upon validation, likely due to better data homogeneity (12, 13).

**Table 1:** Summary of publications describing artificial intelligence application in predicting heart transplant outcomes.

Author	Title	Data source used	Primary outcome	Best model performance	Study conclusion
Oztekin (16)	Predicting the graft survival for heart-lung transplantation patients: An integrated data mining methodology	Unified Network for Organ Sharing (UNOS), 1987–2008	Combined heart-lung transplantation survival, and methods of UNOS database mining	The neural network achieved the highest accuracy of 0.859.	Model uncovered relationships among the survival-related variables. Domain-expert input improved performance.
Delen (7)	A machine learning-based approach to prognostic analysis of thoracic transplantations	United Network for Organ Sharing (UNOS), 1987–2009	Survival time	The support vector machine model with a radial basis Kernel function produced the best fit with anR2value of 0.879 and MSE of 0.023.	Integrated machine learning is more effective in developing the Cox survival models than the traditional methods.
Nilsson (10)	The international heart transplant survival algorithm (IHTSA): A new model to improve organ sharing and survival	International Society for Heart and Lung Transplantation (ISHLT) registry, 1994–2010	1-year mortality	Artificial neural network model had anAUC of 0.650, 95% CI: 0.640–0.655.	The model predicts mortality and also estimates the expected benefit to the individual patient, and donor-recipient compatibility.
Medved (17)	Improving prediction of heart transplantation outcome using deep learning techniques	United Network for Organ Sharing (UNOS), 1997–2011	1-year mortality	International Heart Transplantation Survival Algorithm (IHTSA), based on deep learning, had AUC of 0.654, 95% CI: 0.629–0.679.	The IHTSA model was superior to Donor Risk Index (DRI), Risk Stratification Score (RSS), and Index for Mortality Prediction After Cardiac Transplantation (IMPACT).
Miller (5)	Predictive abilities of machine learning techniques may be limited by dataset characteristics: insights from the UNOS database	Unified Network for Organ Sharing (UNOS), 1987–2014	1-year mortality	The neural network model had the highest AUC of 0.66.	The prognostic abilities of machine learning techniques may be limited by quality of the clinical dataset.
Agasthi (11)	Machine learning helps predict long-term mortality and graft failure in patients undergoing heart transplant	International Society of Heart and Lung Transplant (ISHLT) registry data, 2000–2017	All-cause mortality and graft failure at 5 years after HTx	A gradient-boosted machine model had an AUC of 0.717, 95% CI: 0.696–0.737 for 5-year mortality and 0.716, 95% CI: 0.696–0.736 for graft failure.	This model would likely function as a predictive algorithm to estimate the risk of 5-year mortality and graft failure in each donor–recipient match.
Hsich (15)	Heart transplantation: An in-depth survival analysis	Scientific Registry of Transplant Recipients (SRTR), 2004–2018	Factors that determined survival	AI was used to identify variables that are associated with mortality, classified into early, late, and constant phases.	Transplantation from ECMO should consider end-organ function to reduce early post-transplantation mortality.
Ayers (18)	Using machine learning to improve survival prediction after heart transplantation	United Network for Organ Sharing (UNOS), 2000–2019	1-year survival	The final ensemble model had an AUC of 0.764, 95% CI: 0.745–0.782	Modern ML techniques can improve risk prediction in OHT compared to traditional approaches.
Zhou (13)	Prediction of 1-year mortality after heart transplantation using machine learning approaches: A single-center study from China	Local dataset from Wuhan union hospital, 2015–2018	1-year mortality	Random Forest model achieved the best AUC of 0.801 and gradient boosting machine showed the best sensitivity of 0.271	The model identifies high-risk HTx recipients, informs a personalized therapeutic plan, and reduces organ wastage
Kainuma (19)	Predictors of 1-year outcome after cardiac re-transplantation: Machine learning analysis	United Network for Organ Sharing (UNOS), 2000–2009	1-year survival predictors post heart re-transplantation	Random survival forests-ranked variable importance to evaluate the association with mortality	Short-term survival was related to liver function, and long-term survival was related to obesity and mechanical ventilation.
Mete (14)	Predicting post-heart transplant composite renal outcome risk in adults: A machine learning decision tool	Organ Procurement and Transplantation Network (OPTN), 2000–2019	Dependence on chronic dialysis, GFR < 20 ml/min per 1.73 m2, or having received a kidney transplant	The Random Forest model had AUC of 0.70, 95% CI 0.67–0.74 for the composite primary outcome.	The Model was used to create a validated web-based decision tool for assessing renal outcomes post HTx.
Miller (20)	Temporal shift and predictive performance of machine learning for heart transplant outcomes	United Network of Organ Sharing (UNOS), 1994–2016	1-year all-cause mortality	Random Forest model had an AUC of 0.893, CI: 0.889–0.897.	While AI models can predict transplant mortality, they are limited by temporal shifts in patient and donor selection.
Wang (12)	Comparison of four machine learning techniques for prediction of intensive care unit length of stay in heart transplantation patients	Local data from Wuhan Union Hospital, 2017–2020	Length of ICU stay post heart transplantation	The eXtreme Gradient Boosting (XGBoost) algorithm presented significantly better predictive performance (AUC 0.88).	Using the XGBoost classifier with HTx patients can facilitate precision medicine and best allocation of medical resources.

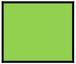
 strong recommendation, 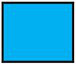 weak recommendation/promising but not ready to implement, and 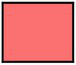 recommended against AI usage, as suggested by the respective paper.

Most studies focused on transplantation survival (*n* = 10 studies), excluding re-transplantation and multiorgan transplantations (*n* = 9 studies). This is in recognition that these subgroups inherently have low frequency, significant patient heterogeneity, and variable management practices. More recent algorithms addressed specific post HTx complications, such as renal dysfunction and ICU stay (12, 14). These tools are key as they provide actionable knowledge that can guide multiorgan transplantation, pre-HTx rehabilitation, and perioperative practices to optimize outcomes (15).

### Post heart transplant management guidance

Seven studies were identified utilizing machine learning or AI and management of heart transplant patients (Table 2). The clinical questions targeted were detection of rejection, cardiac allograft vasculopathy, and guidance of immunosuppression dosing. Models either attempted to automate the steps normally performed by human experts or leverage detailed molecular data to improve sensitivity for early rejection. Two groups described promising AI models for automatic endomyocardial biopsy interpretation; the CRANE model developed by Lipkova and the CACHE-Grader model by Peyster et al., where both reported performance similar to human experts with less variability (21, 22). The CRANE model offers comprehensive biopsy interpretation (rejection type and grade), and it is adaptable to various populations and camera systems allowing for utilization of multicenter data for research and quality control. The CACHE-grader model, on the other hand, applies transcriptome mapping to identify graft rejection earlier than histopathology. Combining automated histopathologic and transcriptomic data will likely advance the accuracy and efficiency of allograft rejection surveillance to a new level (23).

**Table 2:** Summary of publications describing artificial intelligence application in practicing post heart transplant care.

Author	Title	Data source used	Primary outcome	Best model performance	Study conclusion
Hoda (26)	Prediction of cyclosporine blood levels in heart transplantation patients using a pharmacokinetic model identified by evolutionary algorithms	Local institutional data from the Heart and Lung Transplantation Center, University of Vienna Medical School	Cyclosporin blood level	Evolutionary algorithms model had a mean percent error of 8.0 ± 6.7%.	AI model accurately predicted cyclosporine whole blood levels in heart transplant recipients.
Chen (24)	Quantitative 3D analysis of coronary wall morphology in heart transplant patients: OCT-assessed cardiac allograft vasculopathy progression	Local patients at Transplant Center at Institute of Clinical and Experimental Medicine and the Center for Cardiovascular and Transplantation Surgery, Brno, Czech	Coronary artery intimal thickness	Exclusion regions determined by transfer learning using ImageNet network achieved an accuracy of 81.2%.	AI allows quantification of location-specific alterations of coronary wall morphology over time and is sensitive even to very small changes of wall layer thicknesses.
Peyster (22)	An automated computational image analysis pipeline for histological grading of cardiac allograft rejection	Local institutional data at Hospital of the University of Pennsylvania, Cleveland Medical Center, and the Ohio State University Wexner Medical Center	Histopathologic rejection detection	A support vector machine classification model had an AUC of 0.92.	The grader pipeline, derived using intuitive morphological features, can provide expert-quality rejection grading.
Woillard (27)	Tacrolimus exposure prediction using machine learning	local institutional data	Blood concentration of tacrolimus (TAC) following twice daily vs. daily dosing	XBBoost models to estimate TAC blood AUC based on 2 measurements showed mean prediction error close to 0; and root mean square error < 10%.	AI allows accurate estimation of TAC interdose AUC and can be used for routine TAC exposure estimation and dose adjustment.
Lipkova (21)	Deep learning-enabled assessment of cardiac allograft rejection from endomyocardial biopsies	local institutional data from the Brigham and Women’s Hospital	Histopathologic rejection detection, classification, and grading	A neural network (transfer learning from ResNet50 and attention-based multiple instance learning) model had an AUC of 0.962 for detecting allograft rejection.	The AI system showed non-inferior performance to experts and reduced interobserver variability and assessment time.
Piening (23)	Whole transcriptome profiling of prospective endomyocardial biopsies reveals prognostic and diagnostic signatures of cardiac allograft rejection	Local data from the CTOT-03 trial (NCT:0053192) population from University of Pennsylvania and the University of Wisconsin	A gene expression classifier for 0R/1R vs. 2R acute rejection	Random Forest model had an AUC of 0.947.	RNA-seq-based molecular characterization of EMBs shows significant promise for the early detection of cardiac allograft rejection.
Wei (25)	The novel proteomic signature for cardiac allograft vasculopathy	Local patients at University Hospitals Leuven, Belgium	Detection of cardiac allograft vasculopathy (CAV)	XgBoost model showed an AUC 0.71, 95% CI 0.60–0.81.	The proteomic signature might provide insights into CAV pathological processes and help study personalized treatment targets.

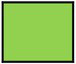
 strong recommendation, 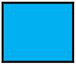 weak recommendation/promising but not ready to implement, as suggested by the respective paper.

The study by Chen et al. offered a deep learning algorithm to analyze high resolution coronary optical tomography images looking for vasculopathic changes (24). Their work offers automatic segmentation of all vessel layers, and it can efficiently detect small changes in coronary architecture on serial measurements. It is novel as it detects vasculopathy early, at a stage where preventative measures might be more effective at avoiding frank graft dysfunction. AI application also allows translation application of molecular markers of graft vasculopathy in the urine, with outcomes nuanced enough to differentiate myocardial injury secondary to rejection vs. vasculopathy (25).

As for medical therapy guidance, two studies developed models to predict cyclosporin and tacrolimus levels (26, 27). Both models used medication history, hepatic and renal functions, infectious status and risk factors, and patient demographics. AI allows plotting drug pharmacokinetics beyond mere trough level, potentially offering more accurate dosing recommendations. While systems demonstrated good performance, they faced the challenges of inability to determine which factors contributed to the outcome, were overfitted, and missed the opportunity to incorporate genomic and transcriptomic variables.

### Post mechanical support outcome prediction

A total of 8 studies utilizing AI and MCS outcomes were identified (Table 3). Of these, one examined VA-ECMO, while all others focused on durable MCS with left ventricular assist devices (LVADs) (28). All studies evaluated survival or adverse events. Two studies utilized AI to identify adverse event profiles, time sensitive analyzes of adverse events, and phenomapping of patient profiles as it relates to the former in the LVAD population (29, 30). Grouping of patients facilitates streamlining evaluation and perioperative care pathways that are closely tailored to the patient’s particular risk profile.

**Table 3:** Summary of publications describing artificial intelligence application in predicting mechanical circulatory support outcomes.

Author	Title	Data source used	Primary outcome	Best model performance	Study conclusion
Kourou (31)	Prediction of time dependent survival in HF patients after VAD implantation using pre- and post-operative data	Local institutional data at the university of Leuven, Belgium	Time dependent survival	Artificial neural network model had an accuracy of 84.5%, sensitivity of 87%, specificity of 82%.	Application of feature selection and prediction algorithms for variable selection significantly improved prediction ability.
Ayers (28)	Predicting survival after extracorporeal membrane oxygenation by using machine learning	Local institutional data at the University of Rochester, NY	Survival	A deep neural network model had an AUC of 0.92.	Improved prediction of survival to discharge for VA-ECMO with ML versus SAVE score.
Bellavia (32)	Usefulness of regional right ventricular and right atrial strain for prediction of early and late right ventricular failure following a LVAD implant: A machine learning approach	Local data from ISMETT center and Papa Giovanni XXIII Hospital, Italy, 2010–2017	Right ventricular failure post LVAD	Naïve Bayes achieved an AUC of 0.78 in predicting Acute RVF, 0.86 in predicting Chronic RVF, and 0.92 in predicting Acute and Chronic RVF.	No single parameter is capable of predicting post LVAD RVF and the combination of risk scores, clinical, imaging and hemodynamic profiles provides the best risk assessment.
Kilic (33)	Using machine learning to improve risk prediction in durable left ventricular assist devices	(INTERMACS), 2006–2016	90-day and 1-year survival	XGBoost algorithm had an AUC of 0.74 for 90 -day mortality, and 0.714 for 1-year mortality.	Machine learning modeling had discriminatory performance, alone or as an adjunct to logistic regression.
Kilic (30)	Machine learning approaches to analyzing adverse events following durable LVAD implantation	ENDURANCE Trial (post-hoc analysis of a prospective, randomized controlled trial)	Adverse events	Hierarchical clustering was used to categorize adverse events.	Machine learning can identify distinct time patterns of post LVAD complications, facilitating research and quality improvement.
Misumi (34)	Prediction of aortic valve regurgitation after continuous-flow LVAD implantation using artificial intelligence trained on acoustic spectra	Local institutional data, Osaka University Hospital, Osaka, Japan, 2015–2017	Development of aortic insufficiency	The ensemble model had an accuracy of 0.91 and AUC of 0.73.	Machine learning trained on acoustic spectra is promising in diagnosing LVAD complications.
Shad (35)	Predicting post-operative right ventricular failure using video-based deep learning	Multicenter (3) registry, United states	Development of post LVAD RV failure	A convolutional neural network model had an AUC of 0.729, 95% CI: 0.623–0.835.	Machine learning can outperform a team of human experts.
Hendren (29)	Phenomapping a novel classification system for patients with destination therapy LVAD	INTERMACS, 2008–2017	survival, adverse events	Unsupervised machine learning clustering analysis.	Machine learning can help identify phenogroups who have differing survival and rates of adverse events post LVAD implantation.

INTERMACS, Interagency Registry for Mechanically Assisted Circulatory Support. LVAD, left ventricular assist device. 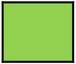 strong recommendation, 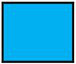 weak recommendation/promising but not ready to implement, as suggested by the respective paper.

The remaining five LVAD studies all evaluated various methods of predicting survival and adverse events post implantation. Consistently, these were determined to have better discriminatory power than human experts given the same task, or conventional risk scoring systems. Collectively, these data suggest that AI techniques can allow for better understanding of patient profiles, timing of MCS related adverse events and can be additive to presently available methods of estimating the risk of post implant mortality. AI also opens new horizons for innovation in device development and surgical techniques, as we can now systematically homogenize study populations to assess the efficacy of each support platform. Ideally, this can then assist in preimplant patient selection as well as post implant monitoring and management to optimize MCS outcomes.

### Post mechanical support management guidance

Guidance of post MCS care has been targeted by only three algorithms (Table 4). The InDetector project successfully implemented deep learning to segment driveline pictures for objective detection and grading of driveline infection (36). This can also be used to follow up response to therapy in the outpatient setting. The algorithm by Maw et al. utilized LVAD log data to diagnose suction events with high success, despite the model overfitting (see below in AI methods) (37). Such physiologic control systems are likely to become more common in the LVAD world, akin to the case of pacemakers, as the large amount of data generated by these devices facilitate AI model training.

**Table 4:** Summary of publications describing artificial intelligence application in guiding mechanical circulatory support practice.

Author	Title	Data source used	Primary outcome	Best model performance	Study conclusion
Aras (36)	InDetector – Automatic detection of infected driveline regions	Local institutional data – University of Arizona, United states	Diagnosis of LVAD driveline infections	The convolution neural network with image augmentation had 93.75% accuracy.	InDetector, a smartphone-based application, allows at-home patients to send images of their driveline to a remote server which used an AI model to classify the driveline region as clean or infected.
Maw (37)	Development of a suction detection algorithm from patient pump data	Local Institutional Data, Medical University of Vienna	Detection of HVAD suction events	The supervised learning algorithm had 92.5% sensitivity and 100% specificity.	The proposed algorithm for suction detection may be used as diagnostic marker, or as a component of an automatic physiologic controller in patients with HVAD pumps.
Topkara (38)	Machine learning-based prediction of myocardial recovery in patients with left ventricular assist device support	INTERMACS, 2008–2017	LVAD explant specifically due to myocardial recovery	Bayesian logistic regression model achieved the highest AUC of 0.824.	Machine learning can be a valuable tool to identify subsets of LVAD patients who may be more likely to respond to myocardial recovery protocols.

HVAD, heartware ventricular assist device. LVAD, left ventricular assist device. INTERMACS, Interagency Registry for Mechanically Assisted Circulatory Support. 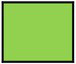 strong recommendation, 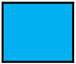 weak recommendation/promising but not ready to implement, as suggested by the respective paper.

One study used patient clinical data to guide post-LVAD medical therapy aiming for myocardial recovery (38). The paucity of similar studies is likely due to the lack of large databanks suitable for AI model development, that follows post MCS management practices along with outcomes. The Interagency Registry for Mechanically Assisted Circulatory Support (INTERMACS) lacks enough granularity on post LVAD care that would be needed for reliable training of AI models to guide medical therapy.

### Summary of AI methods

We noticed a prevalence of utilizing supervised machine learning techniques over unsupervised learning (Supplementary Table 1). A major difference between supervised and unsupervised learning is the availability of labels. Only three studies applied unsupervised ML techniques, while the remaining used supervised ML techniques (7, 29, 30). Each one of these learning techniques encompasses a set of ML algorithms. The choice of the algorithm is governed mostly by the type of data [structured (e.g., medical history), images (e.g., pathology images), longitudinal (e.g., repeated lab measurements), and clinical notes]. The common ML models used to analyze structured data in the reviewed papers were logistic regression, random forest, and eXtreme gradient boosting (XGBoost), likely due to their superior clinical interpretability (see below) (39). There is a notable underutilization of the treasure trove of clinical notes; none of the reviewed papers analyzed clinical notes. Also, we have not yet seen multidomain data integration, such as combining histopathology, echocardiography, and proteomics to diagnose rejection. These models are expected to emerge in the near future *via* transferring AI methods being used in other fields into heart failure cardiology. Of note, the clinical natural language processing methods have been increasingly recognized and matured in healthcare over the past years. Utilizing these methods in heart transplant research may provide insightful information beyond the structured electronic health records.

Deep learning models are more common with unstructured data types such as images and videos, due to their superior abilities in automatically extracting important features from raw data that can help in predicting the outcome. Four of the reviewed papers used transfer learning with convolutional neural networks (CNN). For longitudinal data (e.g., lab measurements collected over time and snapshots of pump data), all the reviewed studies manually extracted fixed-dimensional summary statistics (e.g., minimum, maximum, and standard deviation of the laboratory values in each time frame) from the temporal time series before building the ML model.

Despite the intuitive need for interpretable AI (explanation of why the decision was made) in medical applications, it is relatively underexplored. Only 15 manuscripts described model interpretability. Two of these papers (Shad et al., Lipkova et al.) used saliency maps to highlight the most contributing region of an image to the predicted outcomes (21, 35, 40). Zhou et al. and Wang et al. used Shapely Additive exPlanations (SHAP), which quantifies the contribution of each feature (variable) to the predicted outcome related to a specific instance (12, 13). The rest of the papers used feature importance to explain the outcome of their ML models. Feature importance derivation is done by calculating the model’s performance following the permutation of that feature. If the model performance decreases, then the permutated feature is important. While feature importance and SHAP might look similar, the main difference is that feature importance is centered around the decrease in model performance. In contrast, SHAP confers the magnitude contribution of the feature toward the predicted outcome.

### Summary of model evaluation methods

The area under the receiver operating characteristic curve (AUC) was the primary performance metric used for model evaluation. Accuracy, sensitivity, and specificity were reported inconsistently between studies. Root mean square error (RMSE), mean percent error, and R^2^ were commonly reported when evaluating regression models (e.g., continuous outcome). Overall, moderate to high performance was achieved in the studies for survival prediction after heart transplant, likely due to the availability of large training datasets (UNOS and ISHLT registry). That said, biases (e.g., racial and gender bias) in clinical ML is a key constraint and must be addressed to ensure fairness (41–43). However, only the study conducted by Nilsson et al. investigated the potential bias of the developed model (10). The other studies did not have any bias assessment of the developed AI models.

Model validation enhances confidence in model generalizability and scalability to other medical systems. K-fold cross-validation was used to evaluate and enhance model performance, in which the dataset is split into *K* subsets (folds) *and the model* is trained on *K-1* folds and tested on the remaining *validation* fold. The process is repeated until the algorithm is tested on all folds, and the average performance across all test folds is reported (44). Three studies, in which sample size was less than 60, used leave-one-out cross-validation in evaluating the model’s performance; evaluating the model on one instance / case and training the model using the rest of the cases, iteratively (32, 34, 37). External validation was only used in the study conducted by Lipkova et al. (21). ML predictive performance varies across settings, populations, regions, time, potential biases, and practice patterns, and therefore, there is a need of validating on external sources (45).

## Discussion

**Figure 2:**
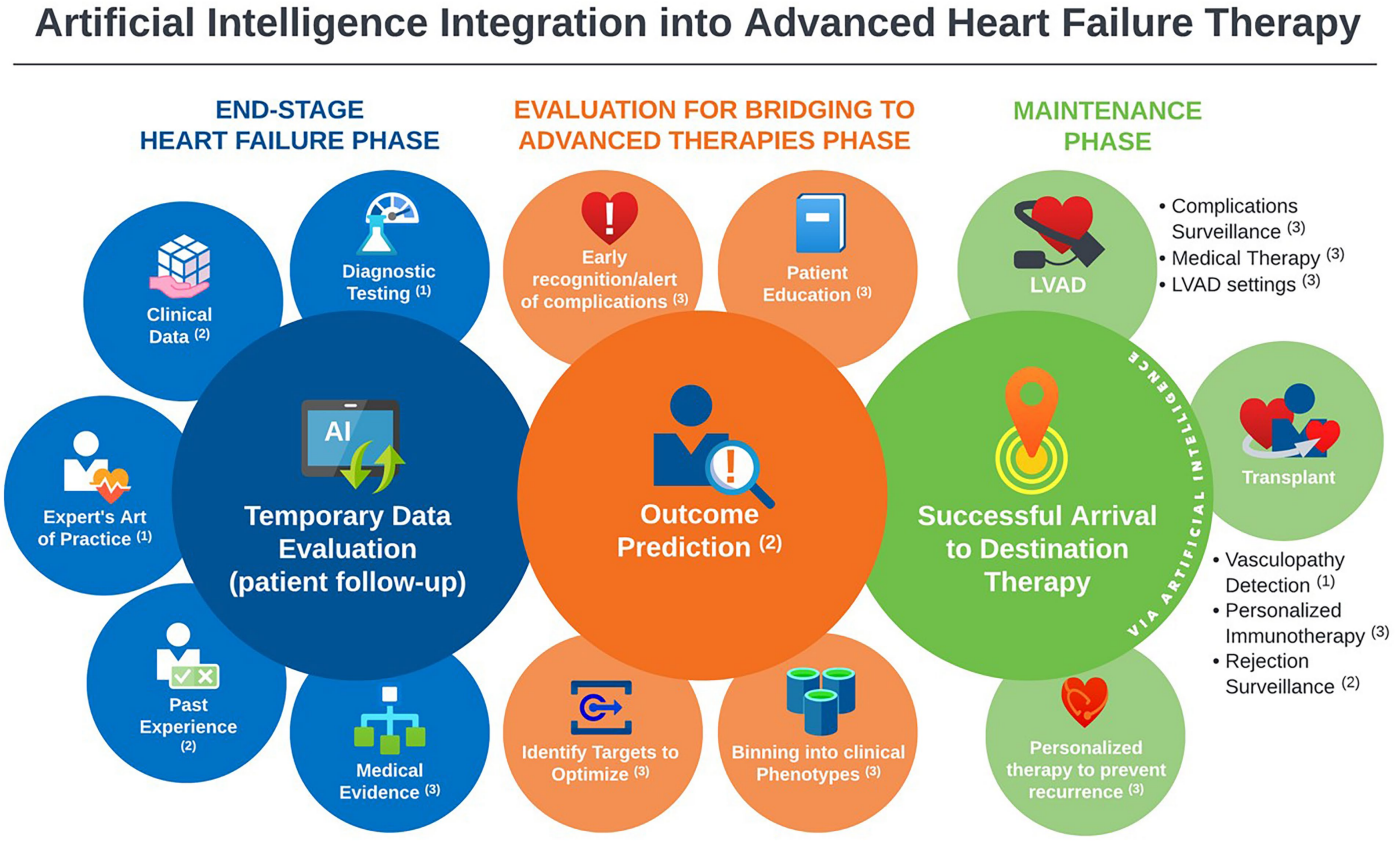
Landscape overview of artificial intelligence applications in advanced heart failure practice, with annotations indicative the level of maturity of the available literature of each application; ^1^Promising, but not yet mature for clinical use. ^2^Good support, ready for prospective testing. ^3^Theoretical potential, but no/negligible support.

In this scoping review, we identified 31 studies addressing the implementation of artificial intelligence in the clinical practice of MCS and heart transplantation published between 2005 and 2022. Most publications focused on outcome prediction using large existing databases. However, there is a rising wave of innovation in AI methods to tackle challenging care aspects that currently consume most post-intervention resources. We found the most mature AI applications in this field: the prediction of survival and significant complications, as well as HTx rejection identification. Moreover, early work is being conducted to further leverage AI power by introducing practical concepts (the art of medicine) into AI systems and integrating multiple biodomains (laboratory data, ultrasound, histopathology) into model conclusions. An important area of active investigation is post HTx graft vasculopathy detection, a highly morbid complication. The capabilities of AI methods demonstrated in the current review have the potential to incorporate medical literature into predictive algorithms, providing personalized guidance to medical management and complication surveillance of HTx and MCS (Figure 2).

Electronic health records contain rich sources of historical and current information that span multiple domains (procedures, diagnoses, medication, and demographics). When harnessed appropriately, it is expected to reveal hidden insights that traditional methods are unable to discover (46). Machine learning (ML) offers flexibility and scalability in assimilating and evaluating large amounts of complex healthcare data. Unlike the traditional statistical methods that focus on inference, ML methods concentrate on prediction by finding patterns in rich and unwieldy data (46). This is evident in complex data formats such as images, time-resolved data series (e.g., LVAD data logs) or wide data matrices (e.g., genomic array). Even though ML can demonstrate superior capabilities in predict patients’ clinical outcomes and risk-stratifying patients according to their clinical and physiological data, it is challenged by the (1) non-explainability of complex algorithms; (2) lack of randomized controlled trials (RCTs) of AI systems, which may not always be feasible; (3) robust evaluation, validation and generalization to various healthcare systems; and (4) identification of biases and unfairness in algorithms. All these factors can hinder the implementation of AI systems in the clinical practice (47, 48).

The domain of Explainable AI (XAI) has emerged as a natural progression to the recent AI developments to increase users’ trust and understanding of the ML black-box systems (49, 50). While some ML models like decision trees, linear models, and attention models are intrinsically explainable, they have lower model accuracies compared to more complex ML models like neural network models (51). However, complex ML models require creating another model to construct explanations, such as using SHAP. The trade-off between intrinsic models and post-hoc models lies between model accuracy and explanation fidelity. Deploying ML in the medical practice requires researchers to put more effort into investigating and evaluating these different explanation techniques to identify which one can best serve health care providers to assess risks and make better decisions.

We cannot overlook the demand to improve the trust and transparency of AI systems used in advanced heart failure, as these decisions affect patients’ quality of life and longevity. Requiring ML systems to (1) justify their decisions/output, (2) enable healthcare providers to take control to identify errors and correct them, and (3) integrate human expert knowledge into models, can contribute to achieving these demands (16). In this scoping review, we found several models that, if validated and implemented, can address vital clinical needs. However, validation was limited by the database availability. The UNOS, INTERMACS, and the ISHLT registry databases are the largest databases available. There is a critical need for data sharing infrastructure that is inclusive of multiple biodomains (imaging, clinical text, electronic heart care system entries, and vital outcomes) to enable generation of accurate ML models that can be validated, meet user’s expectations, and continuously updated to remain current with the clinical practice (52).

As individual systems emerge and become publicly available, pragmatic evaluation for accuracy, gender and ethnic bias and fairness, and safety for medical application becomes challenging. AI programs are recognized as medical devices by the food and drug administration (FDA), with ongoing efforts to govern their clinical application (53). As experts specializing in each particular AI method and application are scarce, unbiased external oversight becomes challenging (54). We have noticed that only one study has external validation. The latter process assures that AI model remain accurate in various settings and are not specifically fitting the population used in the model derivation. “Model waste” can occur where excellent AI models are not clinically applied due to lack of validation (55). Also, there is possibly a publication bias as there is only one manuscript that suggested limited AI benefit (5).

## Limitations

Our scoping review has some limitations. Our search included the 3 major medical databases for feasibility, however, there are many studies published in engineering and bioinformatics journals that may not be indexed in the searched databases. Our results are only up to date as of August 15^th^, 2022. The search criteria may have missed related studies focusing on cardiogenic shock, cardiac imaging, or heart failure patients not on MCS or post HTx, however, with models transferrable to such populations. Second, the strength of recommending the AI algorithm for clinical use was categorized based on the message conveyed to the reviewer by the article discussion and conclusion, which can be subjective. Despite that papers were reviewed by a multidisciplinary team; a more refined approach could be adopted in the future. Lastly, the outcomes of ML algorithms are subject to systematic errors such as biases. Data sources, mathematical approaches, and results interpretation could introduce these biases into the ML pipeline (56). Given that the nature of this review is to highlight the utilization of AI in the field of heart transplantation, the publication bias assessment was not feasible. However, researchers who aim to implement AI applications in the medical field are warranted to assess these biases.

## Conclusion

Our scoping review showed mounting innovation in AI application in MCS and HTx, with largest evidence being for mortality outcome prediction. The past 2 years have witnessed promising models that can guide heart failure cardiologists in HTx donor-recipient matching, allograft surveillance, immunosuppression dosing, and MCS complication screening. While still in infancy, the rate of development and motivation in the community will likely bring AI into heart failure practice in the upcoming 3–5 years.

## Author contributions

MA-A and MM: study design. MA-A, CB, AP, JV, and MM: literature review and data collection. MA-A and BS: tables and figures. All authors have participated meaningfully in the study and approve the final manuscript, interpreted the data, and developed and edited the manuscript.

## Funding

Research reported in this publication was supported by the National Center for Advancing Translational Sciences of the National Institutes of Health under University of Florida and Florida State University Clinical and Translational Science Award UL1TR001427 (MA-A).

## Conflict of interest

The authors declare that the research was conducted in the absence of any commercial or financial relationships that could be construed as a potential conflict of interest.

## Publisher’s note

All claims expressed in this article are solely those of the authors and do not necessarily represent those of their affiliated organizations, or those of the publisher, the editors and the reviewers. Any product that may be evaluated in this article, or claim that may be made by its manufacturer, is not guaranteed or endorsed by the publisher.

## Author disclaimer

The content is solely the responsibility of the authors and does not necessarily represent the official views of the National Institutes of Health.
